# A novel rice grain size gene *OsSNB* was identified by genome-wide association study in natural population

**DOI:** 10.1371/journal.pgen.1008191

**Published:** 2019-05-31

**Authors:** Xiaosong Ma, Fangjun Feng, Yu Zhang, Ibrahim Eid Elesawi, Kai Xu, Tianfei Li, Hanwei Mei, Hongyan Liu, Ningning Gao, Chunli Chen, Lijun Luo, Shunwu Yu

**Affiliations:** 1 Shanghai Agrobiological Gene Center, Shanghai, China; 2 College of Life Science and Technology, Huazhong Agricultural University, Wuhan, China; Washington State Univeristy, UNITED STATES

## Abstract

Increasing agricultural productivity is one of the most important goals of plant science research and imperative to meet the needs of a rapidly growing population. Rice (*Oryza sativa* L.) is one of the most important staple crops worldwide. Grain size is both a major determinant of grain yield in rice and a target trait for domestication and artificial breeding. Here, a genome-wide association study of grain length and grain width was performed using 996,722 SNP markers in 270 rice accessions. Five and four quantitative trait loci were identified for grain length and grain width, respectively. In particular, the novel grain size gene *OsSNB* was identified from *qGW7*, and further results showed that *OsSNB* negatively regulated grain size. Most notably, knockout mutant plants by CRISPR/Cas9 technology showed increased grain length, width, and weight, while overexpression of *OsSNB* yielded the opposite. Sequencing of this gene from the promoter to the 3’-untranslated region in 168 rice accessions from a wide geographic range identified eight haplotypes. Furthermore, Hap 3 has the highest grain width discovered in *japonica* subspecies. Compared to other haplotypes, Hap 3 has a 225 bp insertion in the promoter. Based on the difference between Hap 3 and other haplotypes, OsSNB_Indel2 was designed as a functional marker for the improvement of rice grain width. This could be directly used to assist selection toward an improvement of grain width. These findings suggest *OsSNB* as useful for further improvements in yield characteristics in most cultivars.

## Author summary

Grain weight, including grain length and grain width, is a complex trait, and hundreds of quantitative trait loci (QTLs) were detected in different genetic rice populations. However, only about 10 genes have been isolated and characterized until now. Nine QTLs for grain size were identified by genome-wide association study in a natural rice population. The novel grain size gene *OsSNB* was identified from *qGW7* based on the difference of expression levels between two different varieties with significantly different grain width. OsSNB is an AP2 transcription factor that is negatively regulated grain size. However, OsSNB was found to regulate the transition from the spikelet meristem to the floral meristem and the floral organ development in previous study. Compared to other haplotypes, Hap 3 has a 225 bp insertion in the promoter. Based on the difference between Hap 3 and other haplotypes, OsSNB_Indel2 was designed as a functional marker for the improvement of rice grain width. This can be directly used to assist selection for grain width improvement.

## Introduction

Rice (*Oryza sativa* L.) is one of the most important staple food crops in the world. Grain yield in rice is determined by three components: number of panicles, number of grains per panicle, and grain weight, all of which are complex quantitative traits [1]. Among these traits, the most important trait is grain weight, which is measured as a 1,000-grain weight. The grain weight is largely determined by grain size, which, in turn, includes grain length, grain width, grain thickness, and the degree of filling [1, 2]. These four parameters are positively correlated with grain weight [2].

Over the past 30 years, fueled by the development of DNA markers and genomic sequencing technology, dramatic progress has been achieved in both the mapping and cloning of genes that control grain shape and grain weight in rice. To date, dozens of genes located in main effective quantitative trait loci that control grain shape and grain weight have been isolated by the map-based cloning strategy as well as functionally characterized. Prominent examples are: GRAIN SIZE 3 (*GS3*) [3, 4], *GL3*.*1*/*OsPPKL1* [5–7], *GW5/qSW5* [8, 9], *GS5* [10], *GW2* [11], *GW8/*OsSPL16 [12], THOUSAND-GRAIN WEIGHT 6 (*TGW6*) [13], *GW6a* [14], *GL7/GW7* [15, 16], and GRAIN SIZE ON CHROMOSOME 2 (*GS2*) [17].

Among these QTLs/genes, *GS3* is a major QTL for both grain length and weight, and functions as a negative regulator for grain size [3, 4]. *TGW6* encodes a novel protein with indole-3-acetic acid (IAA)-glucose hydrolase activity that negatively regulates grain weight by limiting the number of cells [13]. *GW5/qSW5* encodes a calmodulin binding protein and acts as a negative regulator for both grain width and grain weight depended on the brassinosteroid (BR) signaling pathway [8, 9, 18]. *GW2*, encodes a RING-type E3 ubiquitin ligase, which also negatively regulates grain width, weight, and yield through negatively regulating cell division in the shell [11].

In addition to these genes that negatively regulate grain size, several genes that positively regulate grain size have also been identified. For example, *GL3*.*1*, encodes a protein phosphatase kelch (PPKL) family Ser/Thr phosphatase, that acts as a positive regulator for grain length [5, 6]. The major QTL *GS5*, which is a putative serine carboxypeptidase [10], and *GW8/OsSPL16*, which is a SBP-domain transcription factor [12], function as positive regulators of grain size, affecting both grain width and weight. *GW6a*, a major QTL for grain weight, encodes a new type of GNAT-like protein that harbors intrinsic histone acetyltransferase activity (*OsglHAT1*). Elevated expression of this gene enhances grain weight and yield by enlarging spikelet hulls *via* both increasing cell number and accelerating grain filling [14]. *GL7/GW7* has been identified as a major QTL for grain length and width, containing a tandem duplication of a 17.1-kb segment at the *GL7* locus. This leads to up-regulation of *GL7*, thus resulting in an increase in grain length [15]. The further dominant QTL *GS2* has been identified, which encodes Growth-Regulating Factor 4 (*OsGRF4*). Increase of *GS2* expression resulted in larger cells and increased numbers of cells, which thus enhances both grain weight and yield [17].

Although the above mentioned genes, that control rice grain size and weight, have been cloned and characterized, several hundred QTLs remain that have been detected by primary mapping to control rice grain shape and grain weight; however, these have not been cloned to date [2]. Isolating candidate genes *via* map-based cloning strategy would be very time consuming, because it needs a long time to develop near-isogenic lines that are required for fine mapping. With the rapid development of sequencing technologies and the continuing decrease in related costs, genome-wide association study (GWAS) has become a powerful tool for the detection of natural variations that account for complex traits [19]. Compared to map-based cloning, GWAS fully utilizes ancient recombination events to identify the genetic loci that underlie traits at a relatively high resolution. Moreover, GWAS requires less research time. GWAS has been successfully applied to the genetic dissection of complex traits in plants, e.g., *Arabidopsis thaliana* [20–23], *Oryza sativa* [24–27], and *Zea mays* [28–31]. Recently, *GLW7*, a major QTL for both grain length and weight, that encodes the plant-specific transcription factor *OsSPL13* was cloned based on a GWAS approach [32]. This example indicates that candidate genes that regulate rice grain size can be quickly discovered *via* the GWAS method.

In this study, GWAS of rice grain width and length was performed in 270 rice germplasms, and several new QTLs and genes associated with grain size in rice were detected. Furthermore, the novel grain width gene *OsSNB* was identified from *qGW7*. The grain width and grain weight of knock out mutant plants were significantly increased and the grain width and grain weight significantly decreased in over-expression transgenic plants. These findings enhance the understanding of the genetic mechanism underlying grain size in rice. These newly reported QTLs/genes of grain size may be useful in future molecular breeding programs aimed at improving grain yield in rice.

## Results

### Descriptive statistics of grain length and width

The natural population (collection 1) showed remarkable segregation for all studied traits (Table 1). Grain length ranged from 4.72 to 10.90 mm with a mean of 8.06 mm in 2011 and from 6.06 to 10.56 mm with a mean of 8.05 mm in 2012. Grain width ranged from 2.45 to 4.23 mm with a mean of 3.28 mm in 2011, and from 2.49 to 4.45 mm with a mean of 3.32 mm in 2012.

**Table 1:** Descriptive statistics of grain length (GL) and grain width (GW) in collection 1.

Traits	Year	valid data	Mean±SD	Range	skewness	kurtosis
GL	2011	235	8.06±0.97	4.72–10.90	0.07	0.58
(mm)	2012	235	8.05±0.85	6.06–10.56	0.66	-0.24
GW	2011	235	3.28±0.41	2.45–4.23	0.06	-0.68
(mm)	2012	235	3.32±0.40	2.49–4.45	0.16	-0.62

Mean±SD, Mean ± Standard deviation

### Genome-wide association study (GWAS) for grain length and grain width

**Fig 1:**
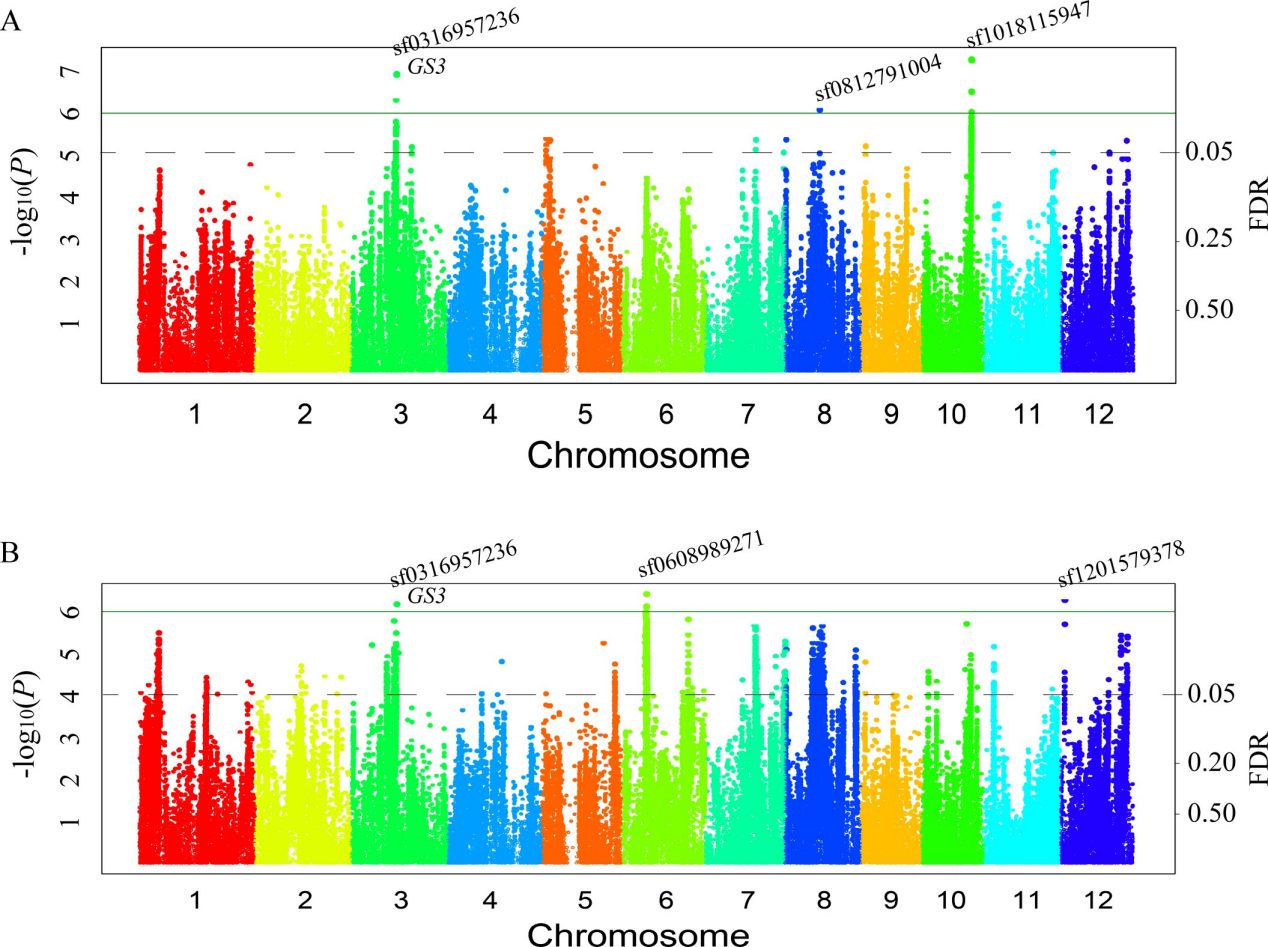
Genome-wide manhattan plots of association mapping for grain length. (A) Grain length in 2011. (B) Grain length in 2012. Note: the nomenclature of SNP includes the information of chromosome and physical position. sf indicates the name of the SNP, and the first two digits indicate the number of the chromosome and the following digits indicate the physical position. The solid green line represents–log_10_(*P*) = 6.0 and the black dotted line represents FDR = 0.05.

Three associated loci (sf0316957236, sf0812791004, and sf1018115947) were detected for grain length at a significance level of–log_10_(*P*) ≥ 6.0 in 2011, which explained 31.81%, 19.88%, and 17.58% of the phenotypic variation, respectively. Furthermore, three associated loci (sf0316957236, sf0608989271, and sf1201579378) were detected for grain length at a significance level of–log_10_(*P*) ≥ 6.0 in 2012, which explained 37.49%, 11.91%, and 11.86% of the phenotypic variation, respectively (Fig 1, Table 2). The association sf0316957236 on chromosome 3 (QTL interval: 16.67–17.00 Mb with *r*^*2*^ of LD > 0.4) was located downstream from the *GS3*, which was located at 16.67 Mb and has a length of about 230 kb. Thus, *GS3* belongs to this QTL interval.

**Table 2:** Summary of GWAS loci of grain length (GL) and grain width (GW).

Loci	QTL	Trait	Year	SNP	Chr	MAF	*P*-value	FDR	PVE (%)
1	*GS3*	GL	2011	sf0316957236	3	0.3	1.25E-07	3.62E-02	31.81
2	*GS3*	GL	2012	sf0316957236	3	0.31	6.76E-07	3.28E-02	37.49
3	*qGL6*	GL	2012	sf0608989271	6	0.39	3.90E-07	3.28E-02	11.91
4	*qGL8*	GL	2011	sf0812791004	8	0.31	8.36E-07	3.62E-02	19.88
5	*qGL10*	GL	2011	sf1018115947	10	0.38	5.78E-08	3.62E-02	17.58
6	*qGL12*	GL	2012	sf1201579378	12	0.14	5.34E-07	3.28E-02	11.86
7	*qGW3*.*1*	GW	2011	sf0313650794	3	0.14	1.67E-07	2.59E-03	0.17
8	*qGW3*.*2*	GW	2012	sf0315013153	3	0.3	1.32E-07	7.32E-04	46.03
9	*qSW5*	GW	2011	sf0505372028	5	0.35	8.40E-15	2.08E-09	45.20
10	*qSW5*	GW	2012	sf0505372028	5	0.39	5.30E-14	1.31E-08	44.79
11	*qGW7*	GW	2011	sf0707318737	7	0.1	4.17E-07	4.81E-03	15.23
12	*qGW7*	GW	2012	sf0707314881	7	0.1	1.71E-08	5.58E-04	16.86

Chr, chromosome; MAF, minor allele frequency; FDR, false discovery rate; PVE, phenotype variation explained by each locus

**Fig 2:**
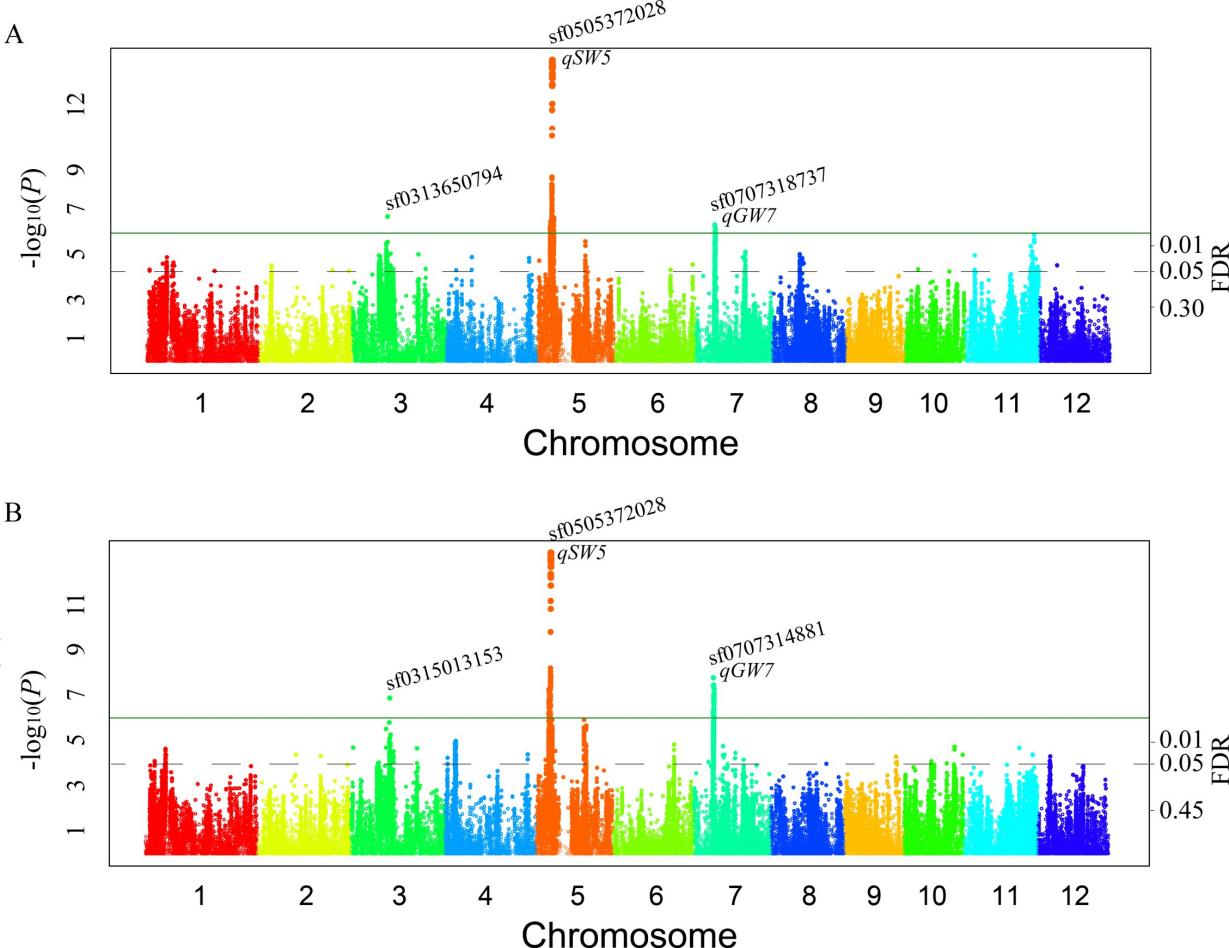
Genome-wide manhattan plots of association mapping for grain width. (A) Grain width in 2011. (B) Grain width in 2012.

Three associated loci (sf0313650794, sf0505372028, and sf0707318737) were detected for gain width in 2011, which explained 0.17%, 45.20%, and 15.23% of the phenotypic variation, respectively. Furthermore, three associated loci (sf0315013153, sf0505372028, and sf0707314881) were detected in 2012, which explained 46.03%, 44.79%, and 16.86% of the phenotypic variation, respectively (Fig 2, Table 2). The *r*^*2*^ of LD = 0.02 between the sf0313650794 and the sf0315013153, thus these are two different association intervals. One associated SNP (sf0505364734 with *P* = 2.32E-07 or 1.37E-07) was located in the promoter of *qSW5/GW5* and was detected for grain width in both 2011 and 2012. The *r*^*2*^ of LD = 0.05 between the sf0315013153 (detected for grain width) and the sf0316957236 (detected for grain length); thus, these are two different association intervals. Additionally, the false discovery rate (FDR) values corresponding to the above nine QTL loci were below 0.05 (Table 2, Figs 1 and 2).

### Candidate gene for grain width was identified in associated loci

**Fig 3:**
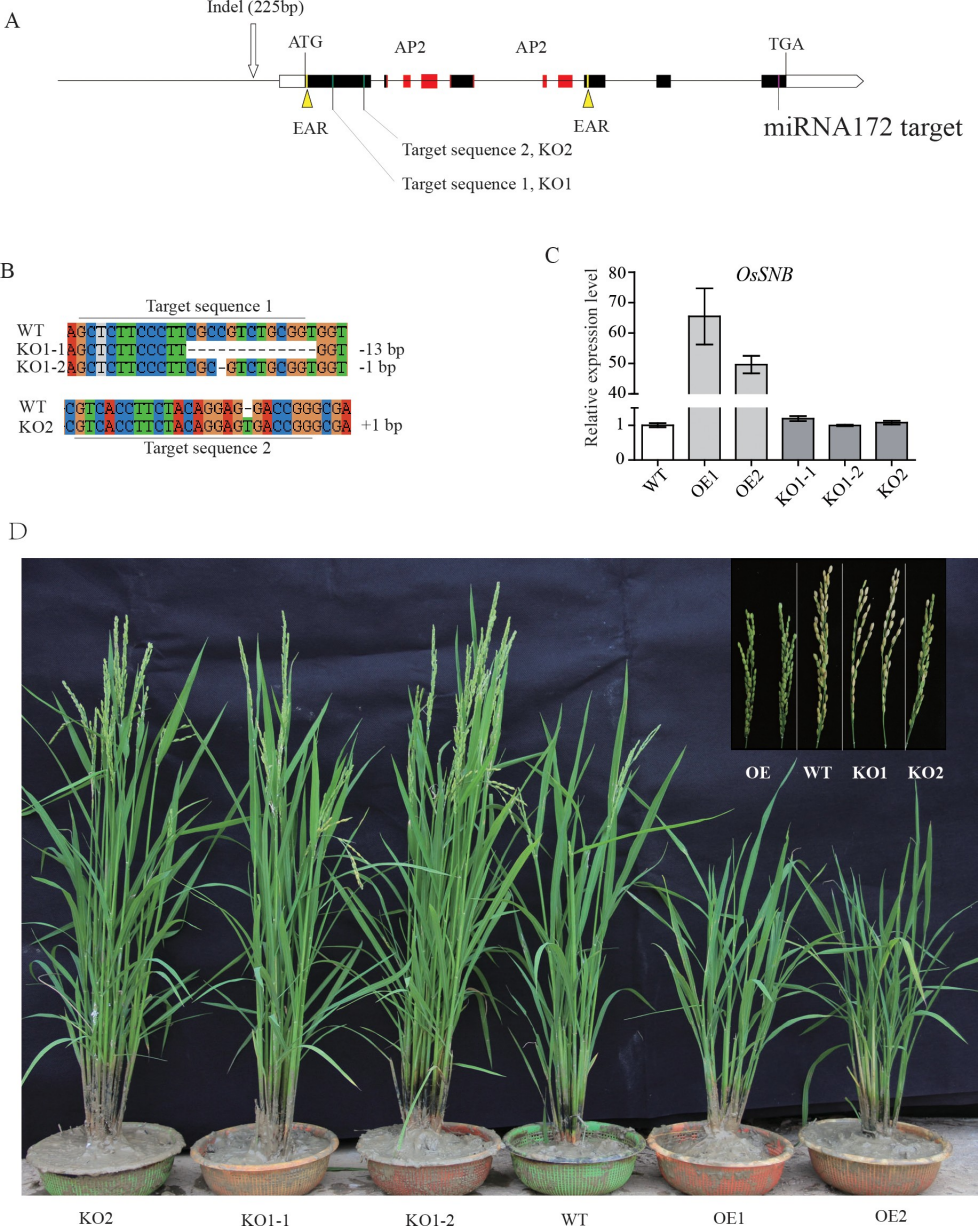
Molecular identification of *OsSNB* transgenic plants. (A) The structure of *OsSNB*. (B) The knockout mutant plants were obtained by CRISPR/Cas 9 technology. (C) Relative expression levels of *OsSNB* in transgenic plants. WT: wildtype; OE1, OE2: overexpression lines; KO1-1, KO1-2, KO2: knockout mutant lines. (D) Phenotypes of transgenic rice plants and WT plants.

**Fig 4:**
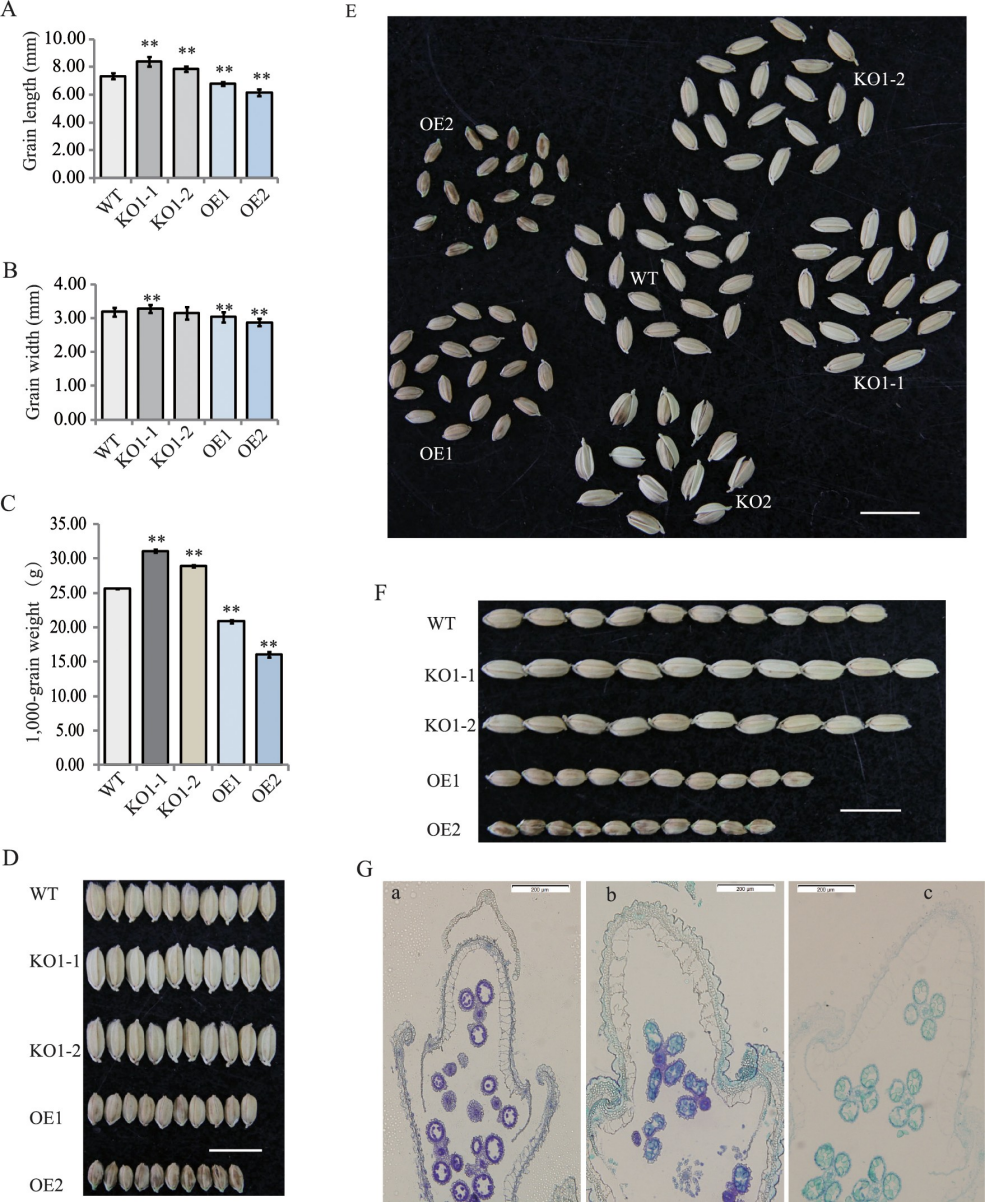
Grain shape was regulated by *OsSNB*. (A) Grain length of transgenic plants. (B) Grain width of transgenic plants. (C) 1,000-grain weight. (D-F) Phenotypes of transgenic plants and wild-type, Bar = 10 mm. (G) Histological analysis of young spikelet in transgenic plants and wild type; OE2 (a), WT (b), KO1-1 (c); Bar = 200 μm.

The SNP sf0707314881 for grain width was identified with a *p*-value of 1.71E-08 in 2012, and the SNP sf0707318737 for grain width was identified with a *p*-value of 4.17E-07 in 2011. The *r*^*2*^ of LD = 0.91 between the sf0707314881 and the sf0707318737; thus, these belong to the same QTL interval (*qGW7*). 219 SNPs were found to be associated with grain width (*P* < 5.00E-06 as threshold line), and 33 putative genes were found in the region of *qGW7* (S1 Table). Two putative genes (LOC_Os07g13170 and LOC_Os07g12730) were differentially expressed (fold change = 1.5100, *P* = 0.0077; fold change = 0.5338, *P* = 0.0171, respectively) *via* microarray data in young panicles in EMATA YIN and *Nipponbare* (S1 Table), with grain widths of 3.06 mm and 3.43 mm, respectively. LOC_Os07g13170 (*OsSNB*), as a candidate drought resistance gene, has been detected in our previous study [33]. Thus, *OsSNB* was cloned from *Nipponbare*, and the over-expression lines were obtained under the 35S promoter *via* transformation. The results showed that the grain width of over-expression lines decreased significantly (Fig 3D, Fig 4). The results indicate *OsSNB* as the candidate gene for grain width in the *qGW7* locus.

### *OsSNB* plays an important role for grain shape

Both sf077544175 and sf077544270 at the promoter of *OsSNB* (LOC_Os07g13170) were detected for grain width with a *p*-value of 1.48E-06 in 2012. *OsSNB* is an AP2 transcription factor and is involved in the development of rice reproductive growth [34, 35].

To test whether *OsSNB* regulates grain width, two gRNA constructs were generated and introduced into the *Nipponbare* to knock out the *OsSNB* gene *via* a CRISPR/Cas 9 strategy [36]. Several homozygous mutant plants were obtained. The mutant plants were identified *via* sequencing of PCR products. The two targets of this gene were knocked out (Fig 3A), and three types of knocked out mutants were identified: KO1-1 (-13 nucleotides), KO1-2 (-1 nucleotide), and KO2 (+1 nucleotide) (Fig 3B). Furthermore, overexpression lines were obtained under the 35S promoter *via* transformation. The transcription levels of overexpression lines increased significantly, showing an approximate 49-fold change compared to wild type (Fig 3C). Compared to wild type, the grain width and 1,000-grain weight of the KO1 mutant lines through first sgRNA vector had increased significantly (Fig 4). Moreover, the grain width and grain length, as well as the 1,000-grain weight of the over-expression lines decreased significantly (Fig 4). The KO2 mutant lines from the second sgRNA resulted in indeterminacy of the floret, which were similar to the *snb* T-DNA knockout mutants reported in a previous study [37]. To obtain the glume cell size of transgenic plants, histological spikelet analysis was conducted. Cross-sections of central parts of the spikelet showed that the glume cells in transgenic KO-1 plants were longer and larger than the corresponding layer in both OE-2 and wild type plants (Fig 4G). These results indicate the involvement of *OsSNB* in cell size regulation during organ development.

**Fig 5:**
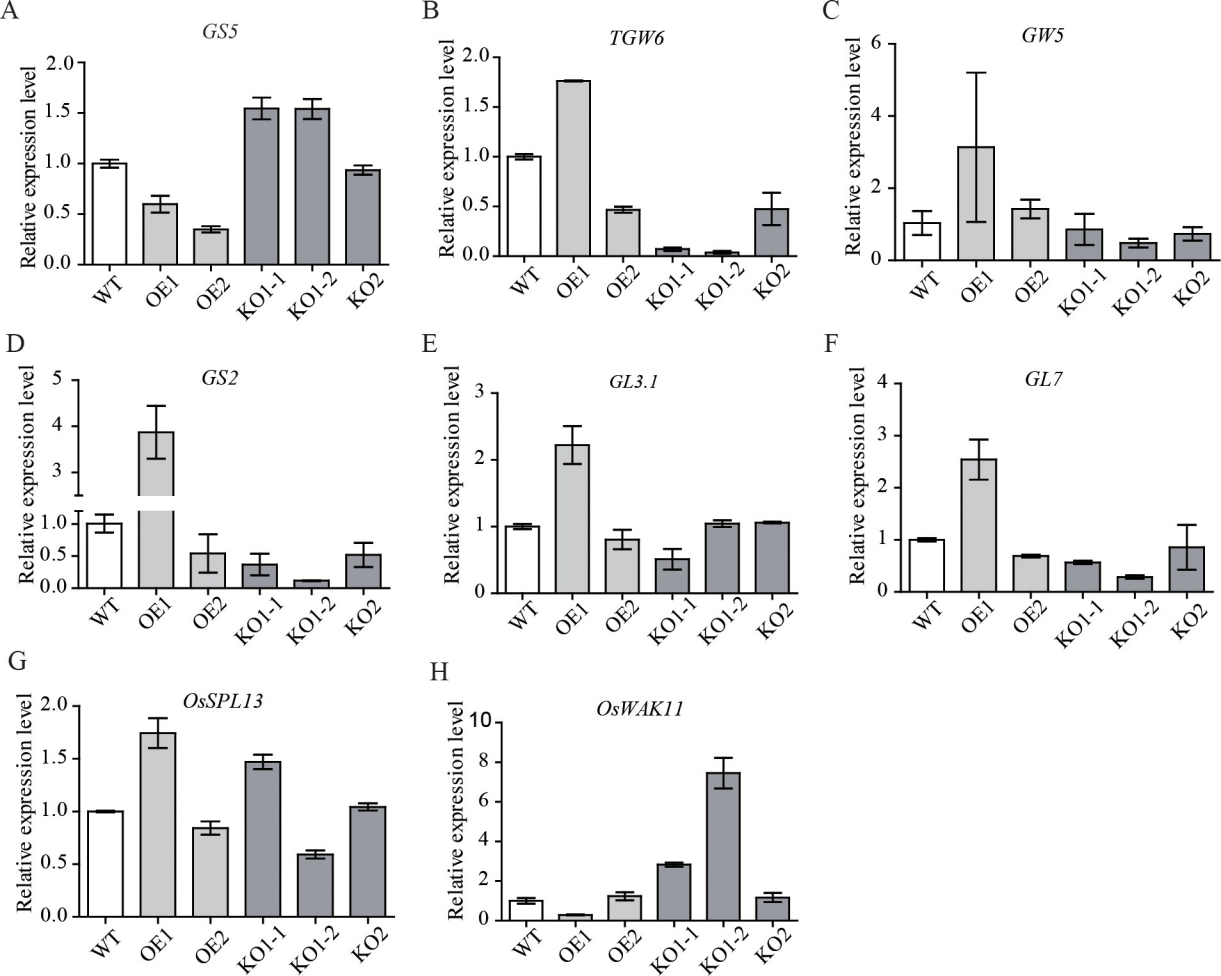
Relative expression levels of known functional genes controlling grain size and grain weight. **(**A-G) Known functional genes controlling grain size. (H) *OsWAK11*, a cell wall-associated receptor kinase. WT: wild type; OE1, OE2: overexpression lines; KO1-1, KO1-2, KO2: knockout mutant lines.

A number of genes that control grain size have been isolated *via* the map-based cloning strategy and have been functionally characterized. To further analyze the regulatory relationship between *OsSNB* and other known functional genes that control grain shape and grain weight, the transcription level of eight known functional genes was analyzed using RT-qPCR (Fig 5). Compared to wild type, the transcription level of *GS5* as positive regulator of grain size was increased in KO1 mutant lines with big grain size, and decreased in over-expression lines with small grain size. The transcription level of *TGW6* acts as a negative regulator and decreased significantly in the KO1 mutant lines with big grain size. These results imply that *OsSNB* may either directly or indirectly regulate the expression of *GS5* and *TGW6*. Additionally, the transcription levels of *OsWAK11* were greatly elevated in the KO1 mutant lines with big grain size. As a cell wall-associated receptor kinase, *OsWAK11* is involved in cell expansion, and its expression is regulated by aluminum, sodium, and copper [38]. However, the expression of these genes remained unaffected in KO2 mutant lines (Fig 5). This suggests that the *OsSNB* fragment with its nuclear localizing signal also has a specific function. Additionally, *OsSNB* can affect both plant growth and development. For example, compared to wild type plants, the over-expression lines of *OsSNB* have delayed flowering (Fig 3D).

### Polymorphism of the *OsSNB* gene

**Fig 6:**
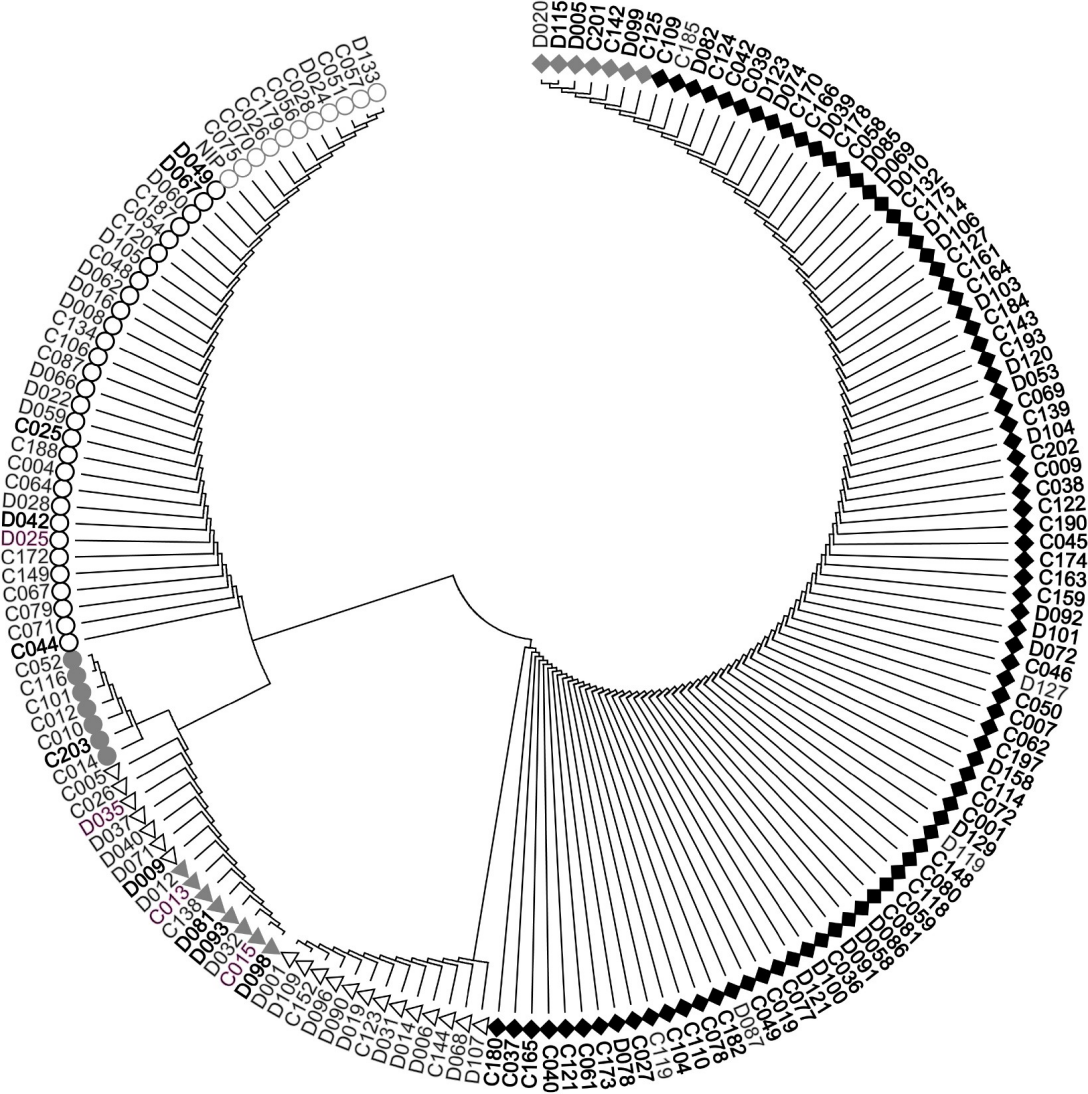
Cluster analysis of *OsSNB*. Black font represents *indica*, gray font represents *japonica*, purple font represents *aus*. NIP = *Nipponbare*.

*OsSNB* was sequenced from the promoter to the 3’-UTR in 168 accessions of the natural population and 65 polymorphic loci were identified (Table 3, S2 Table). The aligned length, including both promoter and open reading frame region of the *OsSNB* gene was 6862 nucleotides. A total of 10 indels and 55 SNPs were detected among the sequenced regions. The number of SNPs exceeded the number of indels. 33 polymorphism loci were located at the promoter region, four and three SNPs were located at extron 1 and extron 10, respectively, 16 SNPs and two indels were located at intron regions, and seven SNPs were located at 3’-UTR. Seven SNPs that were located at the extron region caused non-synonymous mutation. +148 T/C and +149 C/T, result **T**CG (Ser) to **C**CG (Pro), **TC**G (Ser) to **CT**G (Leu); +230 T/C result C**T**G (Leu) to C**C**G (Pro); +242 T/C result A**T**G (Met) to A**C**G (Thr); +3612 G/A result C**G**C (Arg) to C**A**C (His); +3614 T/G result **T**CG (Ser) to **G**CG (Ala); +3674 G/A result **G**CC (Ala) to **A**CC (Thr). No mutation is located at the target loci of miR172. Haplotype analyses were performed based on 65 polymorphism loci of *OsSNB* and the genotype was classified into eight haplotypes (Hap) (Fig 6, S2 Table). Hap 1 and Hap 8 mainly contained the *indica* subpopulation, while Hap 2, 3, 4, 6 and 7 mainly contained the *japonica* subpopulation. Hap 3 has the highest grain width and Hap 1 has the highest panicle length.

**Table 3:** Summary of parameters for the analysis of polymorphism loci in *OsSNB*.

Parameters	Promoter	Coding region	Downstream	Entire region
Total length of amplicons(bp)	2014	3679	1169	6862
Number of all sequence variants (SNPs and indels)	33	25	7	65
Number of SNPs	25	23	7	55
Number of indels	8	2	0	10
Average indels length (bp)	30.875	4.5	-	25.6
Pi	0.00368	0.00257	0.00173	0.00275
Tajima's D	2.28348*	3.72606***	1.40454	3.09934**
Fu and Li's D*	1.83376**	1.83376**	1.15433	2.32478**
Fu and Li's F*	2.41724**	3.10554**	1.47627	3.19789**

*Indicates statistical significance at *P* <0.05

### Nucleotide diversity and selection of *OsSNB*

A total of 6862 bp of genomic region covering *OsSNB*, including a 2014 bp upstream region, 3679 bp of the coding region, and a 1169 bp downstream region, were sequenced in 168 rice accessions. Nucleotide substitutions (SNP) and both insertion and deletion (InDel) variations at the *OsSNB* locus are summarized in Table 3 and S2 Table. The level of polymorphism was highest upstream and a 225 bp InDel at -357 bp was identified upstream. A sliding window of 100 bp at a step size of 25 bp was used to calculate the overall nucleotide diversity (Pi). Pi of *OsSNB* was 0.00275 for all 168 rice accessions, and the coding regions were less diverse than upstream regions. The lowest nucleotide diversity was downstream. The results showed that upstream regions were more diverse than other regions.

Tajima’s D statistic was calculated for upstream, coding region, and downstream, as well as the entire region. Tajima’s D statistic was significant when the entire region, as well as upstream and coding regions were considered. Both Fu and Li’s D* and F* statistics were significant for upstream and coding regions, as well as for the entire region, and not significant for downstream (Table 3). These results indicate that upstream and coding regions may be currently undergoing selection.

### An indel in the promoter region of *OsSNB* is highly associated with rice grain width and was validated as a functional marker

**Fig 7:**
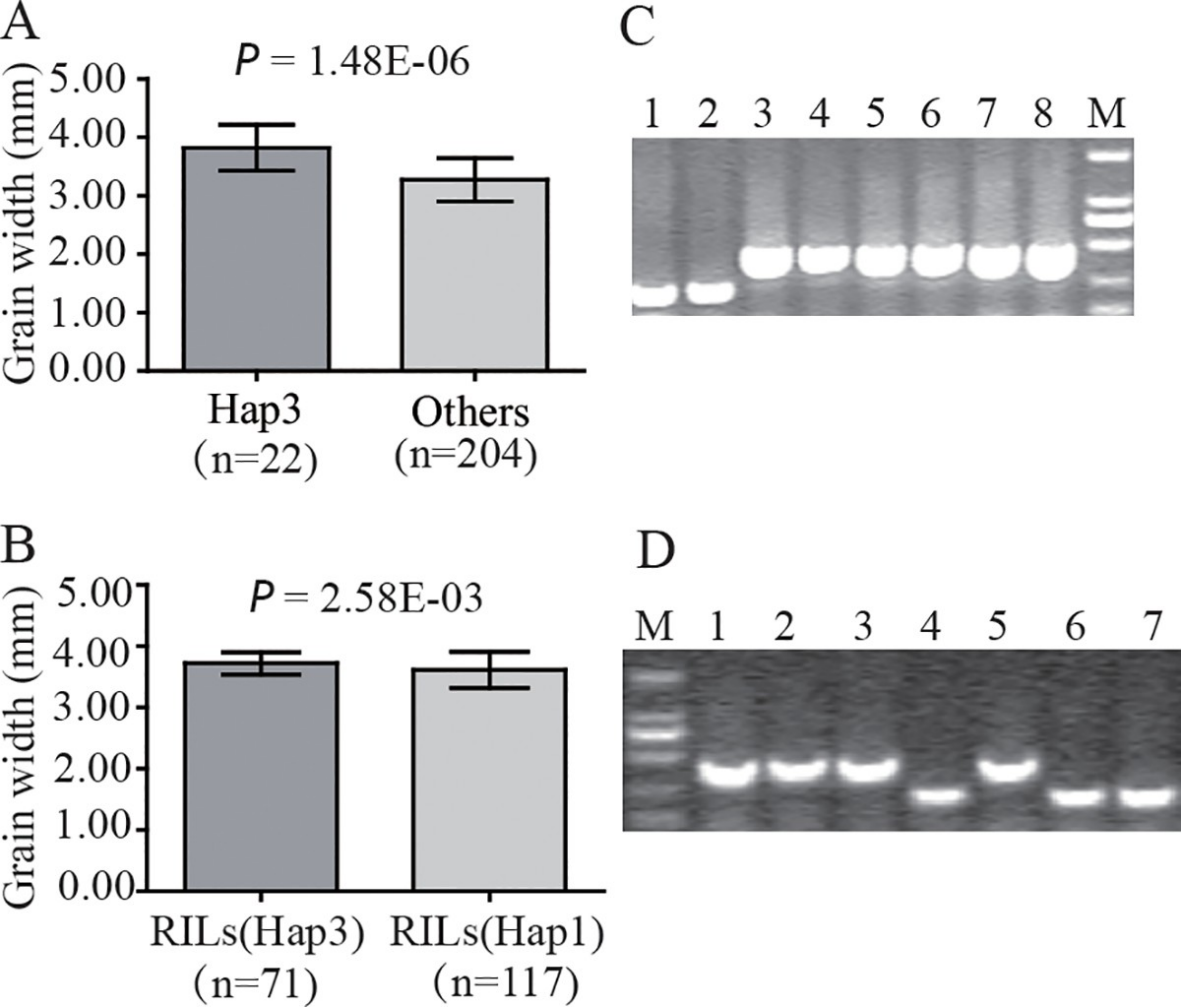
Genotype assay of GWAS population and RILs by the indel marker OsSNB_Indel2. (A) GWAS population. (B) Recombinant inbred lines (RILs). (C-D) Marker assay of OsSNB_Indel2 in natural population and RILs, respectively. Marker (M) indicates the DNA length of 2000 bp, 1000 bp, 750 bp, 500 bp, 250 bp and 100 bp.

Compared to other haplotypes, Hap 3 has a 225 bp insertion located at -357 bp in the promoter region (S2 Table). A significant difference in grain width between Hap 3 and other haplotypes (including Hap 1, Hap 7, and Hap 8) was observed (S2 Table). Thus, an indel marker (OsSNB_Indel2) was developed across a 225 bp insertion/deletion. To test whether OsSNB_Indel2 was a reliable functional marker, the PCR products of the natural population were used for validation by electrophoresis. The results showed that OsSNB_Indel2 can distinguish between different genotypes (Fig 7). The grain widths of 22 rice accessions with 225 bp insertion were 3.82 ± 0.39 mm, the grain widths of 204 rice accessions with 225 bp deletion were 3.28 ± 0.37 mm, the *p* value of Student’s *t*-test was 1.48E-06, and the *p* value of Mann-Whitney *U* test was 3.38E-08. The null hypothesis for this test was that the grain widths are not different between Hap 3 and others. OsSNB_Indel2 can also be used to distinguish the genotypes of recombinant inbred lines (RILs), which were developed from both Zhenshan97B (*indica*) and IRAT109 (*japonica*). The grain widths of 71 recombinant inbred lines with 225 bp insertion were 3.72 ± 0.18 mm. The grain widths of 117 recombinant inbred lines with 225 bp deletion were 3.61 ± 0.30 mm, the *p* value of Student’s *t*-test was 2.58E-03 (Fig 7). These results clearly confirm that this mutation (225 bp insertion or deletion) was highly associated with grain width. Hap 3 was only discovered in *japonica* subspecies, and Hap 3 can be used for marker assisted selection (MAS) for increasing grain width in *indica* subspecies. OsSNB_Indel2 is likely a functional marker for the improvement of rice grain width.

## Discussion

### Novel QTLs were identified by GWAS

Grain shape is controlled by several hundred QTLs and is an important grain yield characteristic in rice. This study detected five significant associated intervals for grain length *via* GWAS in 270 rice accessions, and showed that *GS3* was very closely positioned to the significantly associated interval on chromosome 3 [3, 4]. Four significantly associated intervals for grain width were detected by GWAS in the current study. One associated SNP (sf0505364734) was located on the promoter region of *qSW5* [9]. Two associated loci (sf0313650794 and sf0315013153) were located on chromosome 3, where no grain width QTL/gene was reported in previous studies; thus, these are two novel QTLs. Furthermore, another associated region on chromosome 7 was a novel locus for grain width, which was far away from the functional genes *GL7/GW7* [15, 16] and *OsSPL13* [32], located on chromosome 7. Compared to previous studies, many QTLs for grain size were quickly mapped to smaller intervals in this study. These QTL intervals were more conductive to marker-assisted selection in breeding. Furthermore, smaller QTL intervals enable easier identification of candidate genes. Additionally, gene expression profiling data of predicted genes in the QTL interval can be used to assist in identifying the most promising candidates.

### Mechanism of *OsSNB* controlling grain size

OsSNB is a nuclear protein, that contains two highly conserved AP2 domains [37] and two ETHYLENE-RESPONSE FACTOR Amphiphilic Repression (EAR) motifs (Fig 3A). The transcription level of *OsSNB* is most strongly expressed in the newly emerging spikelet meristems, as well as expressed in other tissues, including shoot, root, sheath, leaf, and seed [37]. In previous studies, *SUPERNUMERARY BRACT (SNB)* was reported to regulate the transition from the spikelet meristem to the floral meristem and to regulate the floral organ development in rice [35, 37], while inhibiting the flowering time in rice by suppressing the expression of *Ehd1* [39]. Overexpression of *rSNB* without the miR172 target site creased branches and spikelets in panicle and RNAi inhibitor of *OsSNB* was the opposite [34], which was similar to the KO1 knockout mutants that had decreased branches and spikelets in this study (Fig 3D). The *snb* T-DNA knockout mutants showed that the transition from spikelet meristems to floral meristems was delayed, which resulted in indeterminacy of the floret [37]. This phenotype was also found in KO2 mutant plants in the present study. *OsSNB* is one of the target genes of miR172, over-expression of miR172 also caused loss of spikelet determinacy and floral organ abnormalities, similar to *snb* T-DNA knockout mutants in rice [40]. Furthermore, no significant difference was found in grain size between wild type and KO2 mutant plants. This indicates that the peptide with 130 amino acid length at the N-terminal is possible playing a competitive role to normal *OsSNB* protein like miR172, because the KO2 mutant still retains a fragment of the nuclear localizing signal. However, further investigations are required to understand other possible roles of *OsSNB*.

**Fig 8:**
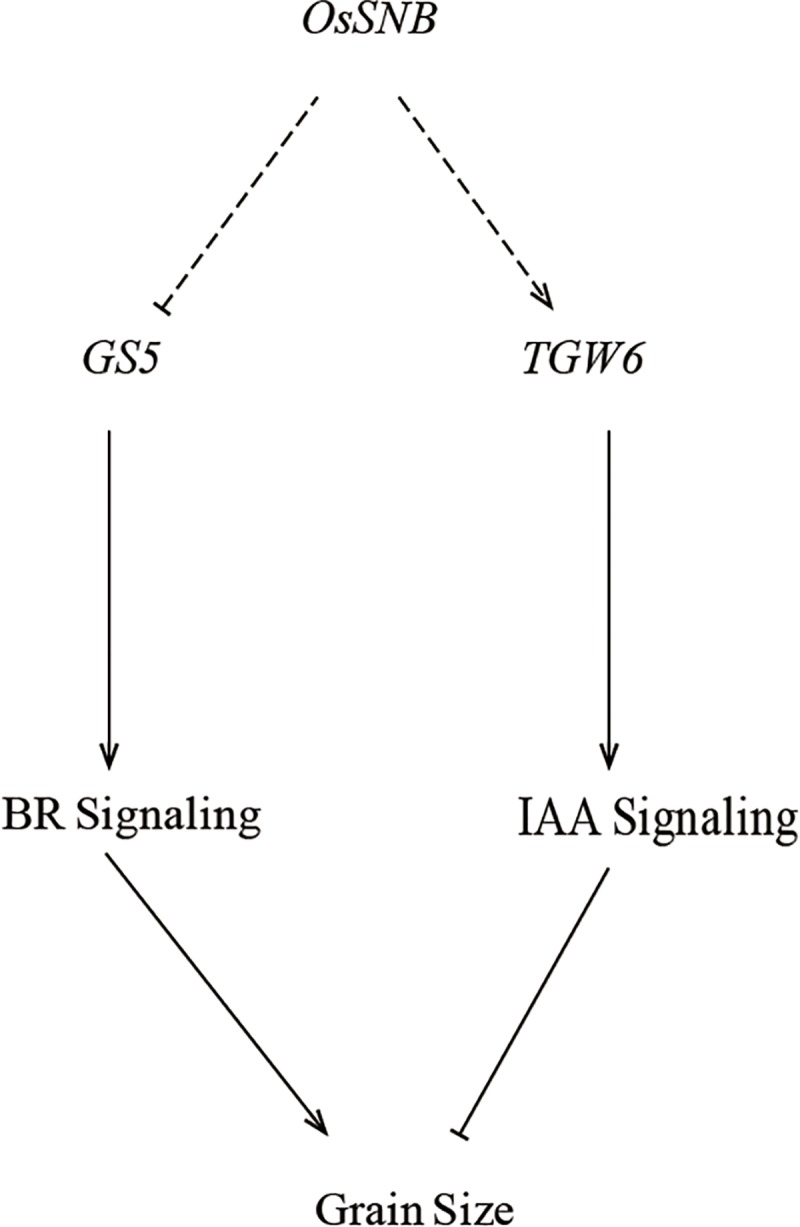
The hypothetical molecular mechanism of *OsSNB* regulation of grain size.

It also remains unclear whether *OsSNB* regulates grain size. It has been shown to regulate the transition from spikelet meristem to floral meristem and to regulate the floral organ development [37]. This study confirmed that *OsSNB* has a novel function, and can regulate grain size. Compared to wild type, KO1 mutant plants have increased grain size; however, over-expression of *OsSNB* plants decreased grain size. Histological analysis of spikelets also showed that *OsSNB* knockout could promote cell enlargement in glumes (Fig 4). These results confirm that the novel grain size gene, *OsSNB* was identified in *qGW7 via* GWAS. Moreover, *OsSNB* can regulate the transcription levels of *GS5* and *TGW6*, two important known functional genes controlling grain size. As a positive regulator, *GS5* controls grain size by BR signaling through regulating grain width, filling, and weight [10, 41]. *GS5* functions putatively as a positive modulator upstream of cell cycle genes; furthermore, its over-expression may result in an increase in cell numbers by promoting mitotic division [10]. The Nipponbare *tgw6* allele affected the timing of the transition from syncytial to cellular phase by controlling IAA supply and limiting both cell number and grain length. Loss of function of the Kasalath allele enhanced grain weight *via* pleiotropic effects on source organs and leads to significant yield increases [13]. These results implied that *OsSNB* may regulate cell number and size and thus affect grain shape through simultaneous BRs signaling and IAA signaling simultaneously (Fig 8). *OsSNB* has been reported to control inflorescence architecture and the establishment of floral meristem in rice [35, 37], and inhibited flowering time in rice by suppressing expression of *Ehd1* [39]. Simultaneously, *OsSNB* was reported to be involved in abiotic stress signaling and was induced by both NaCl and drought [33]. This indicated that *OsSNB* plays a multifunctional role in the response to abiotic stresses and the development of component traits of grain yield.

### Polymorphism analysis of *OsSNB* and development of a functional marker for grain size improvement

In this study, *OsSNB* was sequenced from the promoter to the 3’-UTR in 168 rice accessions. A total of 65 polymorphism sites including 10 indels and 55 SNPs were identified. Rice, as a self-pollinating crop, has a lower nucleotide diversity than maize, which is a typical outcrossing crop [42]. The nucleotide diversity of *OsSNB* exceeds that of *OsC1*, and is lower than that of *OsWx* in rice [43]. Neutrality analysis showed that the upstream and coding regions of *OsSNB* may be undergoing selection; however, the values of Tajima’s D and Fu and Li’s D* and F* based on the *Wx* and *OsC1* locus did not significantly differ from neutral expectations. Eight haplotypes at the *OsSNB* locus were identified by haplotype analysis based on polymorphism sites. The rice accessions with Hap 3 have the highest grain width and Hap 3 is only discovered in *japonica* subspecies. Additionally, the grain width of the lines with Hap 3 was confirmed to be longer than that of lines with Hap 1, using OsSNB_Indel2 in RILs population. Thus, Hap 3 can be potentially used to increase grain width in *indica* subspecies. OsSNB_Indel2 was designed as a functional marker for the improvement of rice grain width, which could be directly used to assist selection for grain width improvement. This marker has practical utility in a variety of tests based on a 225 bp insertion or deletion of specific alleles for rice breeders. This simple, robust, and economic marker genotyping method will both simplify and streamline MAS for grain width in rice breeding.

### Conclusion

The associated loci and genes identified in this study highlight the important underlying mechanisms of grain size. Furthermore, the novel grain size gene *OsSNB* was identified *via* GWAS and was functionally characterized. This locus has eight haplotypes, and Hap 3 has important application value in MAS for grain width in rice breeding. These results provide information for dissecting the genetic and molecular basis of grain size and for improving the grain size of rice through molecular breeding in the near future.

## Materials and methods

### Plant materials

Two rice collections were used in this study. Collection 1 comprised of 270 rice germplasms, including four *aus* accessions, 111 *japonica* accessions, and 155 *indica* accessions. This population has previously been used for GWAS of mesocotyl elongation and drought resistance [24, 44]. This collection was used for GWAS analysis. Collection 2 comprised of 200 F_10_ recombinant inbred lines (RILs), which were developed from Zhenshan97B (*indica*) and IRAT109 (*japonica*). This collection was used for molecular marker verification.

### Phenotyping

To measure grain size, Collection 1 was grown in the field at the Baihe experimental station of the Shanghai Academy of Agricultural Sciences (Shanghai, China) in 2011 and 2012. Collection 2 and two parents (IRAT109 and Zhenshan 97B) were grown in the field at the Baihe experimental station of the Shanghai Academy of Agricultural Sciences in 2014, and grown at the Hainan Experimental Station of the Shanghai Academy of Agricultural Sciences (Hainan, China) in 2015.

Dry seeds were evaluated for grain width and length *via* morphology analyzer (http://www.jsjeda.com). Descriptive statistics analysis of the phenotype data was performed by SPSS version 19.0 (IBM).

### GWAS analysis

Genotype data of the natural population were re-sequenced by Hiseq 2000. Paired-end sequence reads were mapped to a rice reference genome sequence of japonica cv. *Nipponbare* (MSU v6.1), and were used for SNP identification, following the procedures described by Wu et al. [24].

Genome-wide association mapping was conducted by the Efficient mixed model Association (EMMA) method using the Genomic Association and Prediction Integrated Tool (GAPIT) software package in R [45]. A total of 996,722 SNP markers (minimum allelic frequency (MAF) ≥ 0.05), distributed on 12 chromosomes, were used for GWAS in this study. 144,995 SNPs, with missing data below 10% in this natural population, were used to calculate kinship among individuals. Principal component analysis (PCA) was used to adjust the population structure. The genome-wide threshold of–log_10_(*P*) = 6.0 was calculated from the formula of “-log10 (1 / effective number of SNPs)”. *r*^*2*^ of LD was calculated by PLINK ver1.07. The variance explained the phenotype by the lead SNP (with the lowest *P* value) and was calculated by comparing the sum of squares of the variance between groups and the sum of squares of the variance of the full model. These procedures have been described by Ma et al. [44]. The original phenotype data for GWAS are provided in Supplemental S3 Table.

### Sequence analysis of *OsSNB*

*OsSNB* was sequenced from the promoter to the 3’-UTR in 168 rice accessions. Sequence alignment was conducted with ClustalX1.83. Cluster analysis was conducted *via* MEGA 6.0 [46]. Allelic diversities were determined by DnaSP5.0 [47], Pi parameters of nucleotide diversity were estimated by the average number of nucleotide differences per site between any two DNA sequences. Tajima’s D [48] and Fu and Li’s D* and F* statistical tests [49] were used to test the evidence of neutral evolution, which were calculated by DnaSP5.0.

### Molecular cloning and transformation of rice

To construct the overexpression vector, the full-length cDNA of OsSNB with CAMV35S promoter was digested with BamHI and BstEII, and then ligated to pCAMBIA1300. For the construction of the Crispr/Cas9 system knockout vector, two *OsSNB* site-specific single guide RNAs (sgRNA1, 5’-gctcttcccttcgccgtctgcgg-3’; sgRNA2, 5’-gtcaccttctacaggaggaccgg-3’) were introduced into pYLCRISPRCas9Pubi-H according to previously described methods [36]. The three vectors were introduced into the *japonica* rice cultivar Nipponbare *via* the *Agrobacterium*. *tumefaciens*-mediated transformation method [50].

### RNA isolation and RT-qPCR

Total RNA was isolated from rice leaves using the TRNzol reagent (TIANGEN, China). cDNA templates were synthesized using Superscript II reverse transcriptase (TaKaRa, Japan) according to the manufacturer’s instructions. Real-time quantitative RT-qPCR was performed on a CFX96 Real-Time PCR system (Bio-Rad, USA) using SYBR Premix Ex Taq (TaKaRa, Japan) according to the manufacturer’s instructions. The rice Actin1 gene was used as endogenous control. All primers are listed in Supplemental S4 Table.

### Histological analysis

Fresh spikelet samples were collected and fixed in FAA solution for 24 h. After dehydrated in a gradient series of ethanol (70% alcohol, 85% alcohol, and 95% alcohol contain 1% eosin Y) once and pure alcohol twice, each step for 1 h, Spikelet samples were transparentized by a different gradient concentration of chloroform; the utilized volume ratio of chloroform and alcohol was 1/5, 2/5, 3/5, 4/5, and pure chloroform. Then, the samples were infiltrated *via* tiny paraffin in chloroform (at least two days) and embedded in paraffin. Furthermore, microtome sections of 10 μm thickness were put on glass slides. Finally, the slides were deparaffinized in 100% xylene prior to staining by toluidine blue 0.05% and observation *via* light microscope.

## Supporting information

S1 Table Selection of putative genes in *qGW7*. (XLSX)

S2 Table Haplotype analysis of *OsSNB*. (XLSX)

S3 Table Phenotype data of the GWAS population. (XLSX)

S4 Table Primers used in this study. (XLSX)
